# Oxygen Gas Prepared at the Ordinary Temperature

**Published:** 1871-01

**Authors:** 


Oxygen Gas Prepared at the Ordinary Temperature.—Prof. Boettger states that when a mixture is made of equal weights of the peroxide of lead and barium and dilute nitric acid of a strength of 9 degrees baume is poured thereon, a current of pure oxygen gas, free from ozone and antozone, is given off abundantly. This mixture of the two peroxides may be kept dry in a stopped bottle for any length of time.

